# Congenital Hydrocephalus and Abnormal Subcommissural Organ Development in *Sox3* Transgenic Mice

**DOI:** 10.1371/journal.pone.0029041

**Published:** 2012-01-26

**Authors:** Kristie Lee, Jacqueline Tan, Michael B. Morris, Karine Rizzoti, James Hughes, Pike See Cheah, Fernando Felquer, Xuan Liu, Sandra Piltz, Robin Lovell-Badge, Paul Q. Thomas

**Affiliations:** 1 School of Molecular and Biomedical Science, University of Adelaide, Adelaide, Australia; 2 Pituitary Research Unit, Murdoch Childrens Research Institute, Melbourne, Australia; 3 Bosch Institute and Physiology, University of Sydney, Sydney, Australia; 4 Kolling Institute of Medical Research and Sydney Centre for Development and Regenerative Medicine, Royal North Shore Hospital, Sydney, Australia; 5 Division of Stem Cell Biology and Developmental Genetics, Medical Research Council National Institute for Medical Research, London, United Kingdom; 6 Department of Human Anatomy, Universiti Putra Malaysia, Serdang, Malaysia; University of Illinois at Chicago, United States of America

## Abstract

Congenital hydrocephalus (CH) is a life-threatening medical condition in which excessive accumulation of CSF leads to ventricular expansion and increased intracranial pressure. Stenosis (blockage) of the Sylvian aqueduct (Aq; the narrow passageway that connects the third and fourth ventricles) is a common form of CH in humans, although the genetic basis of this condition is unknown. Mouse models of CH indicate that Aq stenosis is associated with abnormal development of the subcommmissural organ (SCO) a small secretory organ located at the dorsal midline of the caudal diencephalon. Glycoproteins secreted by the SCO generate Reissner's fibre (RF), a thread-like structure that descends into the Aq and is thought to maintain its patency. However, despite the importance of SCO function in CSF homeostasis, the genetic program that controls SCO development is poorly understood. Here, we show that the X-linked transcription factor SOX3 is expressed in the murine SCO throughout its development and in the mature organ. Importantly, overexpression of *Sox3* in the dorsal diencephalic midline of transgenic mice induces CH via a dose-dependent mechanism. Histological, gene expression and cellular proliferation studies indicate that *Sox3* overexpression disrupts the development of the SCO primordium through inhibition of diencephalic roof plate identity without inducing programmed cell death. This study provides further evidence that SCO function is essential for the prevention of hydrocephalus and indicates that overexpression of *Sox3* in the dorsal midline alters progenitor cell differentiation in a dose-dependent manner.

## Introduction

Congenital hydrocephalus (CH) is a severe medical disorder which has an incidence of 0.1–0.3% of live births [1]. CH is characterised by the abnormal accumulation of cerebrospinal fluid (CSF) and can result in death if not surgically treated using shunt therapy. CSF is produced by the four choroid plexuses (ChP) located in each of the brain ventricles and its rostral to caudal flow is regulated by the coordinated beating of cilia present on ependymal cells that line the ventricular surface. Non-communicating hydrocephalus results from impaired CSF flow within the ventricular system, which in the majority of cases is due to stenosis of the Sylvian aqueduct (Aq), the narrow passage that connects the third and fourth ventricles. CH has a significant genetic component that is estimated to account for up to 40% of cases [2]. X-linked recessive CH associated with stenosis of the Aq (which comprises 5–15% of genetic cases) is the best characterised form of the disorder and is caused primarily by mutations in the *L1CAM* gene [3]. Familial forms of CH with autosomal dominant and recessive modes of inheritance have also been described, indicating the existence of additional causative genes [2]. However, to date, these genes have not been identified.

Of the model systems that have been used to investigate the aetiology of hydrocephalus and CSF homeostasis, the mouse has proven to be particularly useful. Loss-of-function mutations in several genes that are required for ciliary generation, structure or function in ependymal cells have been shown to cause post-natal hydrocephalus [4], [5], [6], [7]. In addition, ChP defects including loss of cell polarity, abnormal morphology and cytoplasmic expansion have been associated with a number of CH mouse models [8], [9], [10]. In recent years, the subcommissural organ (SCO) has also emerged as a major site of CH pathology [11]. The SCO is a small secretary organ derived from prosomere 1, and is located in the dorsal midline of the third ventricle near the dorso-anterior opening of the Aq. Abnormal SCO development in mice with loss-of-function mutations or ectopic/overexpression of transgenes is also frequently associated with CH [10], [12], [13], [14], [15], [16].

The primary secretory product of the SCO is the glycoprotein SCO-spondin, which polymerises to form Reissner's fibre (RF), a long threadlike structure that extends caudally through the Aq into the spinal cord. Immunological blockage of RF generation results in stenosis of the Aq and subsequent hydrocephalus [17], indicating that RF is critical for maintaining CSF flow through the Aq. Together, these studies point to a model in which RF generated by the SCO maintains patency of the Aq thereby preventing hydrocephalus [10], [12], [13], [14], [15], [16]. However, the causal link between SCO dysfunction and CH has not been adequately resolved as some genetic mouse models of CH with SCO dysplasia also have ciliary and/or ChP pathology [8], [10], [12] or a lack of overt Aq stenosis [8], [16]. Additional CH mouse models with restricted SCO pathology are therefore required to resolve this controversy.

*Sox3* is an X linked transcription factor gene belonging to the *Sry*-like HMG box family that is expressed in most stem/progenitor cells in the developing central nervous system (CNS) of vertebrates [18]. Duplication and mutations of *SOX3* in humans are associated with X-linked Hypopituitarism, a male-specific syndrome characterised by hypothalamic-pituitary axis dysfunction and variable intellectual disability [19], [20], [21]. *Sox3* null mice also exhibit pituitary hormone deficiencies as well as abnormalities of the hippocampus, cortex and hypothalamus, indicating that SOX3 function is evolutionarily conserved [22].

Several studies indicate that *Sox3* acts as a context-dependent regulator of differentiation. Ectopic expression of *Sox3* in the genital ridge of transgenic mice and rearrangements of the *SOX3* locus in humans are associated with XX male sex reversal due to activation of the testis differentiation pathway [23]. In zebrafish, gain-of-function and loss-of-function experiments indicate that *Sox3* is necessary and sufficient for neural plate formation [24], [25]. In addition, enforced expression of Sox3 in chick neural tube progenitor cells inhibits their differentiation [26], [27]. Together, these studies indicate that SOX3 has an important role in generation and maintenance of neural stem/progenitor cells in vertebrate embryos. However, SOX3 function in the developing mammalian brain remains poorly understood at the cellular level.

Here we show that *Sox3* is expressed throughout SCO development in the mouse and that its overexpression in the dorsal midline of the diencephalon, including the SCO, induces CH in a dose-dependent manner. In particular, *Sox3* overexpression blocks the development of the SCO primordium, leading to a failure of RF generation and inhibition of diencephalic roof plate identity.

## Results

### Dose-dependent CH in *Sox3* transgenic mice

**Figure 1:**
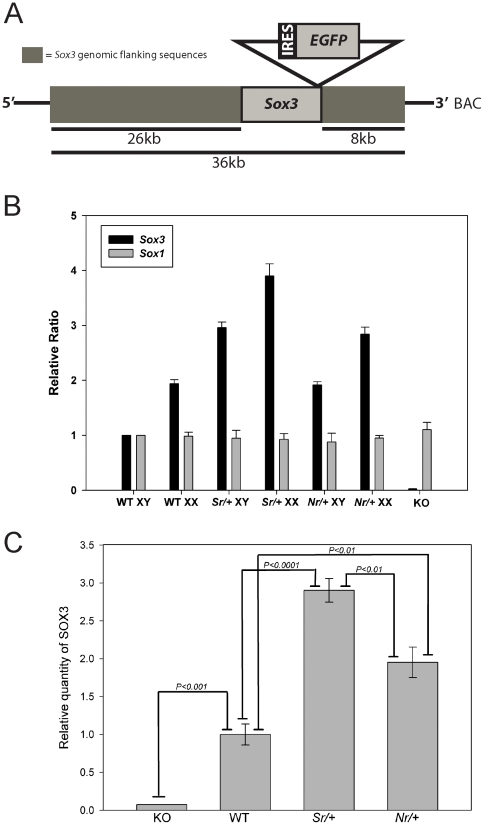
Structure, copy number and expression of the *Sox3* transgene. **A:** Schematic representation of the transgene showing the *Sox3* coding sequence (which is contained in a single exon) and the *IRES*-*EGFP* reporter cassette and flanking genomic sequence (not to scale). IRES indicates the internal ribosome entry site. **B:** qPCR analysis of transgene copy number in the *Sr/+* and *Nr/+* lines demonstrating that they contain 2 and 1 copy of the transgene, respectively. *Sox3* null (KO) genomic DNA was used as a negative control [22]. *Sox1* quantification was performed as an endogenous autosomal control. **C:** Quantitative fluorescence Western blot analysis showing a 2–3-fold increase in SOX3 protein levels in 10.5 dpc *Sr/+* and *Nr/+* embryos.

**Figure 2:**
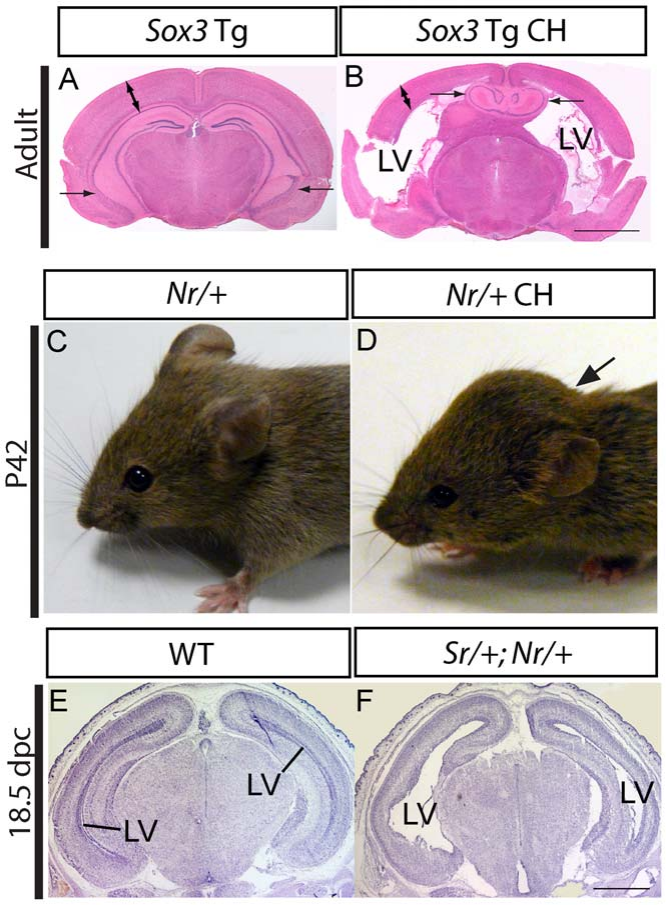
*Sox3* transgenic mice displayed CH. **A,B:** H&E stained coronal section of adult *Sox3* transgenic mouse brain without (A) and with (B) overt hydrocephalus. Note the expanded lateral ventricles (LV), thinning of cerebral cortex (double-headed arrow) and hippocampal deformation (arrows) in the CH founder. **C,D:** *Nr/+* adult mice showing dome-shaped cranium (arrow) due to overt CH (D) and normal cranial morphology (C). **E,F:** Nissl stained 18.5 dpc coronal brain sections of wild type (E) and *Sr/+;Nr/+* (F) 18.5 dpc embryonic brains. Note the expansion of the lateral ventricles (LV) in the transgenic embryo (F). Scale bar: 2 mm (A–B) and 1 mm (E–F).

To investigate the phenotypic consequences of *Sox3* overexpression *in vivo*, we generated transgenic mice by pronuclear injection of a murine *Sox3* genomic fragment containing 34 kb of flanking sequence (Fig. 1A). Two of five transgenic founders/lines developed features of CH including a dome-shaped cranium and enlargement of the lateral ventricles (Fig. 2A,B). To investigate the developmental and molecular basis of this phenotype, additional transgenic mice were generated using a modified transgene in which an IRES-EGFP reporter cassette was inserted into the *Sox3* 3′UTR as a means to detect transgene expression (Fig. 1A) [23]. Six of the twenty founders developed severe hydrocephalus with marked dorsal extension of the cranium, which resulted in death at 6–8 weeks. Lines were only able to be established from two of the twenty *Sox3* transgenic founders. The *Nr* (non-sex reversing) line contained one copy of the transgene and the *Sr* (sex reversing) [23] line contained two copies (Fig. 1B). Quantitative Western blot analysis of 10.5 days post coitum (dpc) embryos indicated that SOX3 levels were ∼2-fold and ∼3 fold higher than wild type in the *Nr/+* and *Sr/+* lines, respectively, consistent with their transgene copy number (Fig. 1C). Neither founder exhibited overt CH but this phenotype was present in 18.7% and 30.6% of *Nr/+* and *Sr/+* adult transgenic descendants, respectively, and in 98.4% of adults that carried both transgenes (referred to hereafter as *Sr/+;Nr/+*; Table 1). Overt CH individuals displayed a domed-shaped cranium due to accumulation of excess CSF prior to fusion of the cranial sutures (Fig. 2C,D). While ventricular expansion was clearly evident in the lateral and third ventricles, the fourth ventricle was not affected in *Sr/+*, *Nr/+* or *Sr/+;Nr/+* mice from 3–5 weeks old, consistent with non-communicating CH (Fig. S1A,B and data not shown). Expansion of the lateral ventricles was observed in *Sr/+;Nr/+* embryonic brains at 18.5 dpc (Fig. 2E,F), confirming the embryonic origin of the phenotype. Together, these data indicate that *Sox3* overexpression during embryonic CNS development causes CH with dose-dependent phenotypic penetrance.

**Table 1:** Dose-dependent penetrance of overt CH in *Sox3* transgenic mice.

Genotype	n	Expected Frequency (%)	Frequency (%)	Percentage with overt hydrocephalus
*Sr*/+	963	50	44.8*	30.6
*Nr*/+	900	50	47.6	18.7
*Sr*/+;*Nr*/+	364	25	17.3∧	98.4

• and ∧: Significantly fewer progeny were present at weaning (*p = 0.030, ∧*p* = 0.001; Chi Square test).

Table showing the penetrance of overt CH in *Sr/*, *Nr/+* and *Sr/+;Nr/+* pups at weaning (approximately 3 weeks).

### Increased dosage of *Sox3* leads to SCO dysplasia

**Figure 3:**
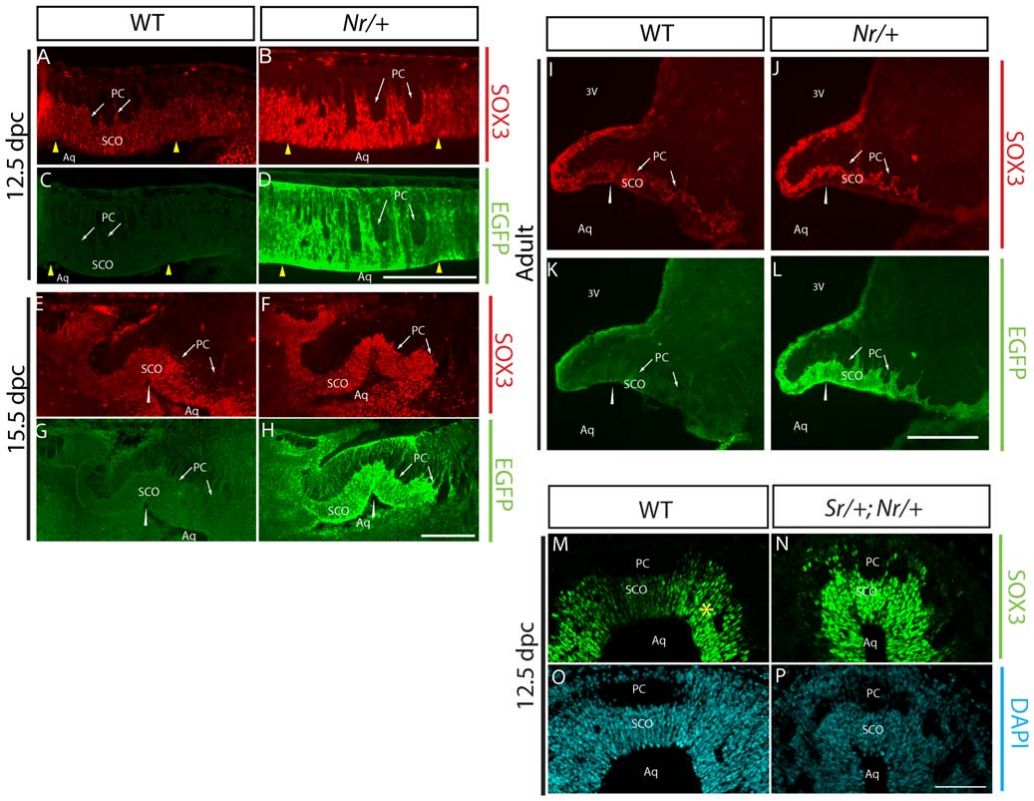
Endogenous *Sox3* and the *Sox3* transgene are expressed in the developing and mature SCO. **A–H:** Sagittal sections of 12.5 dpc (A–D) and 15.5 dpc (E–H) wild type (A,C,E,G) and *Nr*/+ (B,D,F,H) embryos immunostained for SOX3 (A,B,E,F) and EGFP (C,D,G,H). SOX3 is expressed in all cells of the wild type and *Nr/+* SCO. Note the presence of the EGFP signal and higher intensity of SOX3 signal in Nr/+ transgenic sections. **I–L:** Mid-sagittal sections of adult wild type (I,K) and *Nr*/+ (J,L) brains showing expression of SOX3 and EGFP. **M–P:** Coronal sections of 12.5 dpc wild type (M,O) and *Sr/+;Nr/+* transgenic (N,P) SCO tissue. In wild type embryos, a lower level of endogenous SOX3 was detected in the SCO primordium in comparison with periluminal cells flanking the SCO (asterisk in M). This difference in SOX3 signal intensity was not observed in *Sr/+;Nr/+* embryos (N). Arrows: PC (posterior commissure). Yellow arrowheads: points of invagination of the SCO primordium. White arrowhead: mesocoelic recess. In A–L, anterior is to the left and dorsal to the top. Scale bar: 200 µm.

Phenotypic analyses of other CH mouse models have indicated that the SCO has an important role in maintaining CSF flow through the Aq and that abnormal development of this organ is associated with CH [10], [12], [13], [14], [16]. We therefore investigated whether the endogenous *Sox3* gene and the *Sox3* transgene were expressed in the SCO during development and at postnatal stages in the *Nr/+* and *Sr/+* lines using SOX3- and transgene-specific (EGFP) antibodies. In wild type embryos, the SCO primordium is first evident at ∼11.5 dpc as a strip of columnar epithelial cells at the dorsal midline of prosomere 1. Robust expression of SOX3 was detected throughout the SCO primordium at this stage (data not shown) and was maintained in the incipient organ during progenitor cell proliferation at 12.5 dpc (Fig. 3A). By 15.5 dpc, at which time most cells in the SCO have assumed their characteristic tall columnar differentiated morphology, SOX3 expression was detected in all cells (Fig. 3E). SOX3 continued to be expressed in the mature SCO at 18.5 dpc (Fig. S1C) and this expression persisted into adulthood (Fig. 3I). Notably, SOX3 expression was markedly lower in the SCO region compared with the periluminal neural progenitor cells flanking the SCO at 12.5, 15.5 and 18.5 dpc (n = 3 for each stage; Figs. 3M,O, S2A,B,E,F). Analysis of EGFP expression in *Nr/+* and *Sr/+* embryos revealed that the *Sox3* transgene was also expressed in the dorsal midline and periluminal neural progenitor cells flanking the SCO. In fact, EGFP was readily detected in the *Nr/+* SCO primordium at 11.5 (data not shown), 12.5 and 15.5 dpc and was maintained at 18.5 dpc and adult SCO (Fig. 3D,H,L). As a consequence, the level of SOX3 expression at the SCO was elevated to a level that was comparable to that of the periluminal neural progenitor cells flanking the SCO (Figs. 3M,N, S2C).

**Figure 4:**
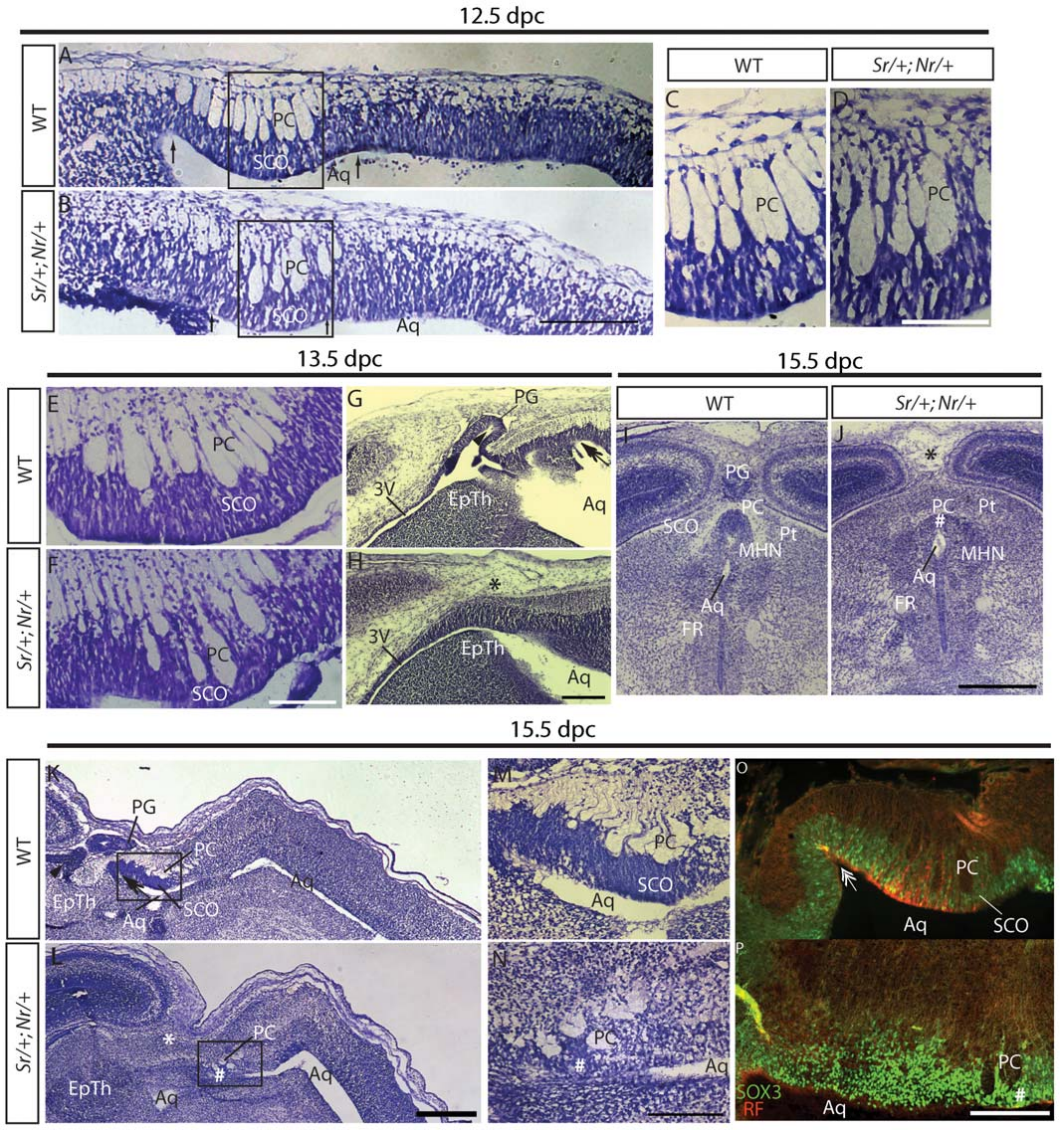
Defective SCO development in *Sr/+;Nr/+* embryos from 13.5 dpc. **A–D:** Nissl stained sagittal sections of 12.5 dpc wild type (A,C) and *Sr/+;Nr/+* (B,D) SCO primordia showing similar morphology and cell body density. C and D are boxed regions in A and B, respectively. **E–H:** Nissl-stained sagittal sections of 13.5 dpc wild type (E,G) and *Sr/+;Nr/+* (F,H) SCO region. Note the thinner SCO primordium and lack of pineal recess in the *Sr/+;Nr/+* embryo. **I–N:** Nissl-stained sagittal (I,J) and coronal (K–N) sections of 15.5 dpc wild type (I,K,M) and *Sr/+;Nr/+* (J,L,N) SCO region. Note the severely disrupted PC and absence of pseudostratified ependymal layer of the SCO in the *Sr/+;Nr/+* embryo. M and N are boxed regions in K and L, respectively. **O–P:** RF (red) and SOX3 (green) immunostaining of 15.5 dpc sagittal wild type (O) and *Sr/+;Nr/+* (P) sections. *Sr/+;Nr/+* transgenic embryos maintain SOX3 expression in the dorsal midline but fail to generate RF. Arrows point to the SCO invagination in the roof plate and the double-headed arrow to the mesocoelic recess. The hash sign indicates the SCO remnant. The asterisk indicates the approximate position where the pineal gland should develop in *Sr/+;Nr/+* embryos. The arrowhead indicates the pineal recess. EpTh: epithalamus. MHN: medial habenular nucleus. Pt: pretectum. FR: fasciculus retroflexus. PC: posterior commissure. In A–H and K–P, anterior is to the left and dorsal to the top. Scale bars are 100 µm (C–F, M,N), 200 µm (A,B,G,H,K,L) and 1 mm (I,J).

Next, we investigated whether SCO development was compromised in *Sox3* transgenic mice. Histological analysis of hydrocephalic P42 *Nr/+* mice revealed striking hypoplasia of the SCO (n = 3), which was reduced to a thin layer of poorly organised flattened cells lining the dorsal limit of the third ventricle subadjacent to the posterior commissure (PC; Fig. S1G–I). While these data suggest that SCO development was impaired by overexpression of SOX3, it is also possible that these defects could be secondary to increased intracranial pressure associated with CH. Consequently, we examined embryonic SCO development, in particular from 12.5 to 15.5 dpc during which the SCO undergoes rapid proliferation and differentiation [28]. This analysis was performed using compound hemizygous *Sr/+;Nr/+* animals because of the near complete penetrance of CH (Table 1). At 12.5 dpc, *Sr/+;Nr/+* embryos had formed a thickened, pseudostratified columnar epithelium at the dorsal midline which closely resembled stage-matched wild type SCO primordium (n = 5; Fig. 4A–D). However, slight irregularity of the PC fibres was evident in some *Sr/+;Nr/+* embryos (data not shown). By 13.5 dpc, the SCO primordium of *Sr/+*;*Nr/+* embryos was markedly thinner with fewer elongated SCO cells compared with wild type embryos (n = 3). In addition, the pineal recess and pineal gland, midline structures that form immediately anterior to the SCO, were not present in *Sr/+*;*Nr/+* embryos (n = 3; Fig. 4E–H). At 14.5 dpc, the SCO was barely discernable by histological analysis and, unlike wild type tissue, densely-stained tall columnar cells were absent from most of the SCO region (n = 2; Fig. S1J,K). At 15.5 dpc, tall columnar cells were only found at the very posterior limit of the *Sr/+;Nr/+* SCO region (n = 3; Figs. 4I–N, S1L–O). The PC was grossly disorganised and the pineal gland and pineal recess were absent. At 18.5 dpc, the dorsal midline of *Sr/+;Nr/+* transgenic embryos contained a small remnant of SCO-like tissue. Although this structure contained cells with elongated morphology, they were less densely compacted than in the wild type SCO and their nuclei were not localised basally. Apart from this region, the PC was also missing in 18.5 dpc *Sr/+*;*Nr/+* embryos (n = 3; Fig. S1P,Q). In contrast to the dorsal midline, other diencephalic structures ventral to the SCO that express the transgene (data not shown), including the ventral thalamus, dorsal thalamus, epithalamus and pretectum, appeared to be morphologically normal (data not shown).

To assess SCO function, we studied SCO secretory function using an anti-RF antibody [8]. RF-positive cells were present in the wild type SCO at 15.5 and 18.5 dpc but were not detected in the dorsal midline of stage-matched *Sr/+*;*Nr/+* embryos (n = 3; Fig. 4O,P and data not shown). Together, these data indicate that the SCO primordium appears initially to form in *Sr/+;Nr/+* embryos but subsequently fails to develop into a functional mature organ.

To investigate the mechanism that leads to SCO dysplasia, we compared apoptotic cell death in wild type and *Sr/+;Nr/+* SCO primordia. Intriguingly, despite the apparent atrophy of the transgenic SCO, we were unable to detect any active caspase-3 positive SCO cells in 12.5–14.5 dpc *Sr/*+;*Nr*/+ and wild type embryos (Fig. S2I–P, data not shown). In contrast, active caspase-3 positive cells were readily detected in the trigeminal ganglia (data not shown), a tissue that has previously been shown to express this apoptotic cell marker between 12.5 and 14.5 dpc [29].

**Figure 5:**
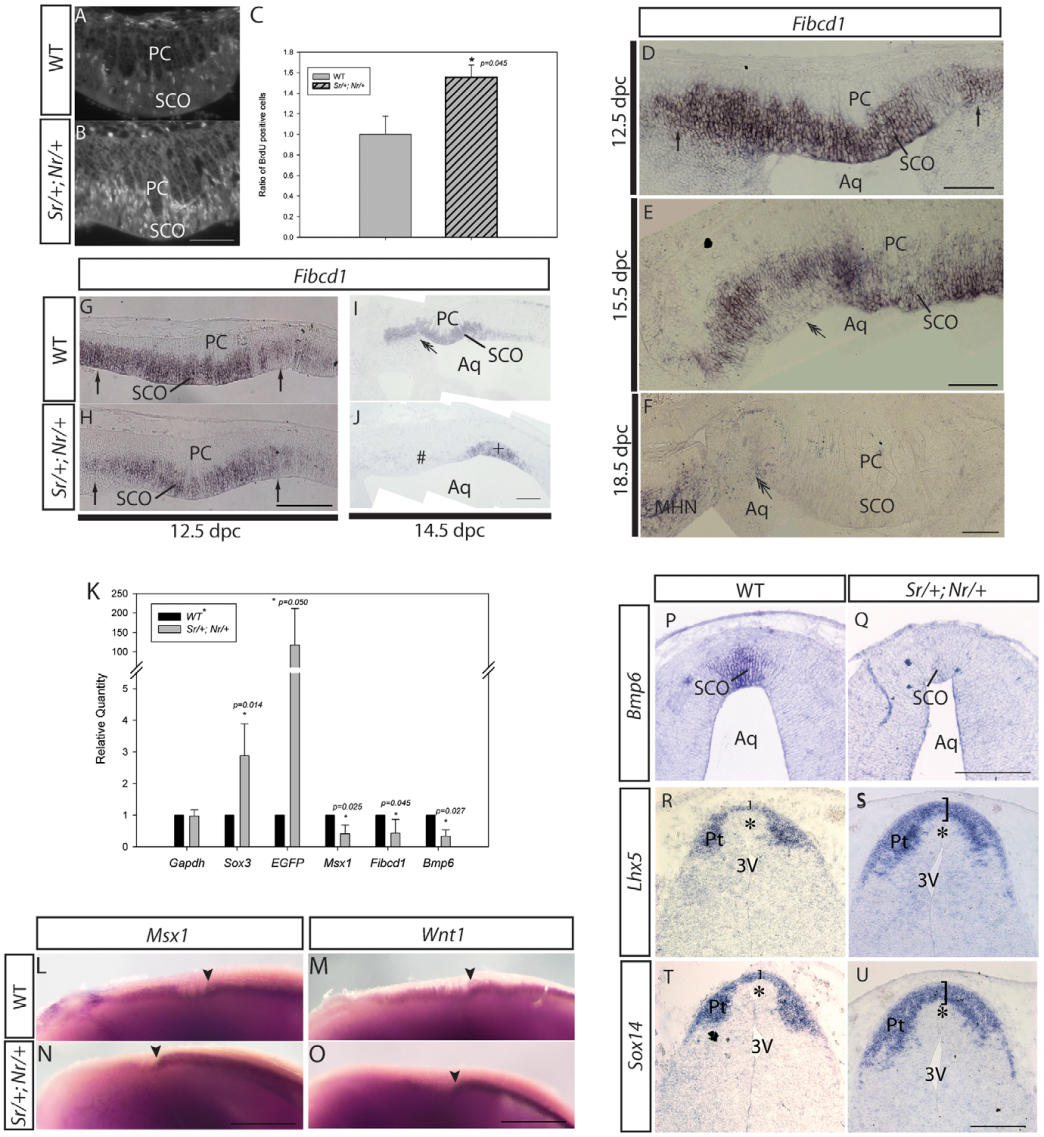
Molecular and cellular defects in *Sr/+;Nr/+* SCO development at 12.5 dpc and 14.5 dpc. **A,B:** Saggital section of 12.5 dpc wild type (A) and *Sr/+;Nr/+* (B) SCO region showing immuno-positive BrdU cells. **C:** Quantitation of BrdU showing a significant increase in proliferation in the *Sr/+;Nr/+* SCO. **D–F:** Section *in situ* hybridisation analysis of *Fibcd1* expression in 12.5 dpc (D), 15.5 dpc (E) and 18.5 dpc (F) SCO. **G,H:** Sagittal section *in situ* hybridisation analysis showing decreased *Fibcd1* expression in the *Sr/+;Nr/+* (H) SCO compared with wild type SCO (G) at 12.5 dpc. **I,J:** In situ hybridisation analysis of wild type (I) and *Sr/+;Nr/+* (J) 14.5 dpc sagittal sections showing robust *Fibcd1* expression in the wild type SCO but not in the dysplasic *Sr/+;Nr/+* SCO region (hash). A small group of *Fibcd1* positive cells were present at the posterior limit of the *Sr/+;Nr/+* SCO region (indicated by the plus sign). **K:** qRT-PCR analysis of SCO marker expression in 12.5 dpc wild type and *Sr/+;Nr/+* SCO biopsies. **L–O:** Whole mount *in situ* hybridisation showing *Msx1* (L, N) and *Wnt1* (M,O) expression at the dorsal midline of wild type (L,M) and *Sr/+;Nr/+* (N,O) embryos at 12.5 dpc. *Wnt1* and *Msx1* expression was lower in *Sr/+;Nr/+* embryos rostral to the posterior limit of SCO (arrowhead). **P, Q:** *In situ* hybridisation analysis of coronal sections from 12.5 dpc wild type (P) and *Sr/+;Nr/+* (Q) embryos showing dramatic downregulation of *Bmp6* expression in the transgenic SCO. **R–U:** *In situ* hybridisation of coronal sections from 12.5 dpc wild type (R and T) and *Sr/+;Nr/+* (S and U) demonstrating the dorsal domain of dorsolateral neuroepithelial cells has expanded ventrally. MHN: medial habenular nucleus. PC: posterior commissure. Arrows point to the SCO primordium invagination. Double-headed arrows indicate the mesocoelic recess. In A, B, D–J and L–O, anterior is to the left and dorsal to the top. Scale bars are 100 µm (A–B, D–H), 200 µm (I–J, P–U) and 500 µm (L–O).

Next, we measured the proliferative index of transgenic SCO progenitor cells at 12.5 dpc by BrdU labelling. A previous study has shown that at this stage most SCO progenitor cells have been born but have not yet terminally differentiated [28]. Quantification of BrdU-positive cells demonstrated that the proliferative index of the *Sr/+;Nr/+* SCO was significantly (1.5-fold) higher than stage-matched wild type controls (n = 4; Fig. 5A–C and Fig. S2U–X). These data suggest that the failure of the SCO to mature in *Sox3 Sr/+;Nr/+* transgenic embryos is not due to apoptotic cell death and that *Sox3* overexpression may alter the fate of the cells in the dorsal midline.

Previous analysis of *Sox3* overexpression in the chick neural tube indicated that SOX3 inhibits neural differentiation through maintenance of a progenitor cell state [26], [27]. We therefore hypothesised that *Sox3* overexpression inhibited the differentiation of SCO progenitors, resulting in the persistence of an immature SCO that lacks terminally differentiated elongated cells expressing RF. To test this hypothesis, we required SCO-progenitor specific markers, which, unfortunately, have not been described. To address this, we interrogated the Allen Brain Atlas for genes that are expressed in the incipient SCO prior to differentiation and are downregulated in the mature organ [30]. *Fibrinogen C domain containing protein 1* (*Fibcd1*), which encodes a transmembrane protein that binds to acetylated-glucosamine [31], met these criteria and this was confirmed by *in situ* hybridisation analysis of *Fibcd1* expression in wild type SCO. *Fibcd1* was robustly expressed across most of the SCO primordium at 12.5 dpc (Fig. 5D). Expression was highly variable across the SCO at 15.5 dpc (Fig. 5E) and by 18.5 dpc, *Fibcd1* expression could not be detected in the SCO but was evident in the medial habenular nucleus of the epithalamus anterior to the SCO (Fig. 5F).

In 12.5 dpc *Sr/+;Nr/+* SCO, which appears morphologically normal, *Fibcd1* expression was less robust than wild type SCO with some cells displaying markedly reduced levels of expression (Fig. 5G,H). qRT-PCR analysis of dissected SCO tissue confirmed that expression of *Fibcd1* in the 12.5 dpc *Sr/+;Nr/+* SCO was significantly reduced compared to wild type (Fig. 5K). At 14.5 dpc, *Fibcd1* expression in the *Sr/+;Nr/+* SCO was markedly reduced compared to wild type with only a subset of cells residing at the posterior limit of the SCO expressing *Fibcd1* (Fig. 5I,J). Together, these data do not support the progenitor cell maintenance hypothesis. Indeed, the premature downregulation of *Fibcd1* in some SCO cells as early as 12.5 dpc suggests that the transgenic SCO is initially specified but rapidly loses its molecular identity during early development.

### Overexpression of *Sox3* inhibits formation of the diencephalic roof plate

Previous published data indicate that the establishment of dorsal midline identity in prosomere 1 is critical for SCO development and that *Msx1* and *Wnt1* (both of which are expressed the dorsal midline/SCO primordium) are required for this process [14], [16]. To investigate whether midline identity was perturbed by increased expression of *Sox3*, we analysed *Msx1* and *Wnt1* expression at 12.5 dpc, the latest time-point at which in *Sr/+;Nr/+* embryos displayed a histologically normal SCO. In wild type embryos, *Msx1* and *Wnt1* were expressed across the entire diencephalic and rhombencephalic midline. In contrast, *Msx1* and *Wnt1* expression in the dorsal midline of *Sr/+;Nr/+* embryos was largely restricted to the region posterior to the SCO (n = 3; Fig. 5L–O). qRT-PCR analysis of dissected SCO tissue confirmed that *Msx1* expression was significantly reduced in *Sr/+;Nr/+* embryos compared to wild type (∼2-fold; *p = 0.025*, n = 5; Fig. 5K) and, although not statistically significant, *Wnt1* expression was also lower in 4 of the 5 samples analysed (data not shown). Expression of *Bmp6* in the dorsal midline/SCO region (which is dependent on *Msx1* activity in Prosomere 1) [14] was also significantly reduced in *Sr/+;Nr/+* embryos as shown by *in situ* hybridisation (n = 3; Fig. 5P,Q) and qRT-PCR (3-fold; *p = 0.027*, n = 3; Fig. 5K). These data indicate that development of the dorsal midline is compromised in *Sox3 Sr/+;Nr/+* transgenic embryos.

To investigate whether the dorsal midline cells adopt an alternative fate, we analysed the expression of *Lhx5* and *Sox14.* At 12.5 dpc, these genes are expressed in dorso-lateral neuroepithelial cells of the diencephalon and a thin band of cells spanning the midline (Fig. 5R,T). Interestingly, analysis of *Sr/+;Nr/+* embryos revealed a marked expansion of the dorsal midline expression domain (Fig. 5S,U; n = 3 embryos). Given the loss of dorsal midline marker expression *Sr/+;Nr/+* embryos, these data suggest that the diencephalic roof plate may adopt a lateral neuroepithelial cell fate in response to high levels of *Sox3* expression.

### ChP development is not overtly affected in *Sox3* transgenic embryos

CH is a heterogeneous disorder that can be caused by defects in CNS regions apart from the SCO, including the ChPs, which are the main centres for CSF production. The ChPs are located in the lateral ventricles and the midline of the third and fourth ventricles. Due to the non-communicating nature of the hydrocephalus phenotype in the *Sox3* transgenic model and the retention of CSF within the rostral ventricles of the brain, it is unlikely that the ChP from the fourth ventricle is contributing to the CH phenotype. In 12.5 dpc wild type embryos, SOX3 is not expressed in the ChP epithelium of the third ventricle and is expressed at a very low level in the ChP epithelia within the lateral ventricles. Endogenous SOX3 was not detected in any ChP at 15.5 dpc (data not shown). Weak transgene expression was detected in the ChP primordia in the third and lateral ventricles of *Nr/+* embryos at 12.5 dpc but not at 15.5 dpc (data not shown). However, histological analysis of the lateral and third ventricle ChP at 15.5 dpc (n = 2) did not reveal any abnormal features (Fig. S2 Q–T), indicating that weak transgene expression in these regions did not perturb development of the ChPs.

## Discussion

CH is a severe medical disorder with a significant genetic component. However, for most cases, the causative gene(s) is not known. Here we show for the first time that overexpression of *Sox3* in the dorsal midline of the murine diencephalon causes CH in a dose-dependent manner. This phenotype is associated with a critical failure in SCO development, in which progenitor cells appear to be induced but fail to terminally differentiate into RF-generating cells. These data strongly reinforce the causative link between SCO dysfunction and CH, and indicate that *Sox3* expression levels must be tightly regulated to ensure normal development of the diencephalic roof plate.

It was first proposed by Overholser *et al.* in 1954 that RF is generated by the SCO and that this fibre plays a role in preventing closure of the Aq [32]. This hypothesis is supported by several loss-of-function (*Msx1*, *Rfx4* and *Pax6*) and gain-of-function (*Adcyap1r1* and *En1*) mouse models of CH in which SCO development and/or function is severely compromised [8], [11], [12], [13], [16], [33]. However, in most of these lines, additional pathologies in tissues that are required for CSF production and flow (including the ChP and ependymal cells) have prevented the establishment of a definitive causative link between SCO dysfunction and CH. In contrast, our data indicate that the CH phenotype in *Sox3* transgenic mice is caused specifically by SCO dysfunction alone. In particular, we observed CH in *Sr*/+;*Nr*/+ embryos at 18.5 dpc. This precludes involvement of ependymal motile cilia, which only become functional from P7. *Sox3* overexpressing embryos also lacked histological defects in the ChPs in the lateral and third ventricles. Instead, the CH-related pathology in our model is restricted to the dorsal midline of prosomere 1 from which the SCO is derived. The failure of the SCO to develop in *Sr/+;Nr/+* embryos becomes apparent at 14.5 dpc, just prior to overt hydrocephalus at 18.5 dpc, and therefore cannot be attributed to secondary defects associated with increased intracranial pressure. Furthermore, the fourth ventricle of hydrocephalic *Sox3* transgenic mice is not enlarged, consistent with blockage of the Aq. Together these data strongly support a direct role for the SCO/RF complex in the prevention of CH by maintaining the patency of the Sylvian Aqueduct.

In wild type embryos, *Sox3* is expressed in neural progenitor cells along the entire neuraxis of vertebrate embryos [34] as well as a small subset of terminally differentiated neurons and glia in the postnatal mouse brain [22, P. Thomas, unpublished data]. Here we show that *Sox3* is also expressed throughout SCO ontogeny. The SCO primordium is first apparent at 10.5–11.5 dpc as a thin band of ependymal cells in the diencephalic roof plate, immediately subjacent to the PC [28]. *Sox3* expression at this stage encompasses the entire diencephalic roof plate (including the SCO) and is generally at a lower level in the midline compared with the lateral neuroectoderm. *Sox3* expression in the SCO is then maintained after terminal differentiation and into adulthood (unlike the vast majority of CNS cells). This may reflect a role for SOX3 transcriptional target genes in SCO function and/or maintenance. In keeping with endogenous *Sox3* expression, the 36 kb *Sox3* genomic fragment used as a transgene in this study was expressed throughout SCO development. However, other embryonic CNS regions that express high levels of endogenous *Sox3* including the telencephalon and mesencephalon were negative for transgene expression (K. Lee and P. Thomas, unpublished data), suggesting that regulatory elements for these locations lie outside the 36 kb transgene, consistent with a recent analysis of conserved sequences flanking the *SOX3* locus [35].

An important property of the *Sox3* transgenic model is that overexpression of *Sox3* in the dorsal diencephalic midline causes SCO dysplasia and CH via a dosa-sensitive mechanism. Single (*Nr/+*) and dual (*Sr/+*) copy embryos develop CH in only 18% and 30% of cases, respectively, and in general have normal SCO morphology. In contrast, compound hemizygous embryos with 3 copies of the transgene develop overt CH with extremely high penetrance (over 98%) and invariably exhibit abnormal SCO development. These data suggest that *Sox3* expression levels in the dorsal midline must exceed a critical threshold in order to exert an effect on SCO development. The phenotypic consequences of *Sox3* overexpression are first evident in the immature organ at 12.5–13.5 dpc indicating that the *Sox3* threshold effect occurs in SCO progenitor cells and blocks their differentiation into terminally differentiated RF-expressing cells. We tested three possible mechanisms that could account for this defect. Firstly, gain-of-function associated with *Sox3* overexpression could lead to increased cell death in SCO progenitors. We found no evidence of elevated apoptosis in the SCO region from 12.5 to 14.5 dpc arguing against this possibility. Indeed, cell proliferation was higher in the dorsal midline of *Sr*/+*;Nr*/+ embryos which was more consistent with a change in cell fate. Secondly, overexpression of SOX3 could prevent differentiation of *Fibcd1*-expressing SCO progenitors. This possibility is supported by a previous study which showed that overexpression of SOX3 in chick neural tube inhibits differentiation of neural progenitors [26], [27]. Importantly, we did not detect an overabundance of *Fibcd1*-expressing cells at 14.5 dpc in *Sr*/+*;Nr*/+ embryos suggesting that SCO progenitor cells do not persist. A third possibility is that SCO progenitors are induced but change their fate as a consequence of elevated *Sox3* expression. Notably, *Fibcd1*is expressed at 12.5 dpc in *Sr/+;Nr/+* embryos indicating that SCO progenitors are induced. However, even at this early stage of SCO development, *Fibcd1* expression was weaker in *Sr/+;Nr/+* embryos consistent with a change in fate. Expression of *Msx1* across the dorsal midline (which is required for normal SCO development [14]) was also reduced. In addition, dorsal midline expression of *Wnt1* and *Bmp6* was also downregulated in *Sr/+;Nr/+* embryos. These genes have previously been implicated in SCO development and are likely to provide important patterning information for the dorsal diencephalon [36]. Together these data suggest that SCO progenitor cells are initially specified but are diverted from their normal developmental fate through overexpression of *Sox3*. It is also possible that *Sox3* overexpression at the dorsal midline negatively impacts on the induction of roof plate cells such that fewer *Fibcd1*-expressing cells are generated at 12.5 dpc.

While the ultimate fate of *Sox3* transgene-expressing cells in the SCO region is not currently known, it is possible that they may differentiate into cell type(s) that normally express relatively high levels of SOX3. One such cell population is the lateral neuroectodermal cells in the diencephalon. Indeed, the (elevated) level of SOX3 expression in the SCO primordia of *Sr/+;Nr/+* embryos is indistinguishable from that of periluminal neural stem/progenitors located in adjacent dorso-lateral region. The higher SOX3 dosage may thus divert the SCO progenitors from a dorsal midline fate towards a neuroepithelial fate. This possibility is supported by the expansion of *Sox14* and *Lhx5* expression domains in the dorsal midline of *Sr/+;Nr/+* embryos at 12.5 dpc. Further gene expression studies are required to address this issue.

The molecular mechanism by which overexpression of *Sox3* alters midline cell fate remains to be identified. However, given that SOX3 can function as a transcriptional activator [19], [23], [26], [37], it seems likely that elevated expression of SOX3 target genes may directly influence differentiation. As SOX3 targets are not currently known, global gene expression and ChIP-seq analysis will be required to explore this hypothesis further. Alternatively, or in addition, higher expression of SOX3 in the dorsal midline may reduce canonical Wnt signalling by direct interaction with nuclear β-catenin [37], [38], which may have a significant impact on dorsal patterning of the diencephalon [36], [39], [40], [41]. Given that the *Sox3* transgene is expressed across the entire dorsal midline of the diencephalon, the impact of SOX3 overexpression may not be restricted to the SCO but could also extend to other regions of the roof plate. This hypothesis is supported by the abnormal morphology of the pineal gland in *Sr/+;Nr/+* embryos and further studies are underway to determine the mechanism by which *Sox3* overexpression affects roof plate induction and differentiation across the entire Prosomere 1 region.

Finally, it is important to consider how SCO dysfunction in the *Sox3* transgenic mouse model relates to CH in humans. Although the human SCO atrophies during early childhood [42], [43], the presence of SCO abnormalities in human fetuses with hydrocephalus suggest that SCO function may be required for CSF homeostasis prior to birth [44]. We have shown previously that duplication of *SOX3* in 46 XY males is associated with CNS defects and congenital hypopituitarism, [19], [21], [45]. However, these patients (which are exceedingly rare) do not exhibit CH. This is perhaps not surprising as *SOX3* duplication in humans is genetically most similar to the *Nr*/+ transgenic line which also contains one additional copy of the *Sox3* gene and has relatively low penetrance of the CH phenotype. Further studies are therefore required to determine the relationship between elevated *SOX3* dosage and CH in humans. To this end, familial X-linked CH cases in which *L1CAM* mutations have been excluded or considered unlikely would appear to be a good starting point [46], [47].

## Materials and Methods

### Animal Ethics

Animal experiments were approved by the University of Adelaide Animal Ethics Committee (S-074-2008). All studies were conducted in accordance with the principles of animal replacement and reduction and experimental refinement. Animals were monitored daily for evidence of CH and, if distressed, were culled immediately by cervical dislocation by an experienced investigator/animal technician.

### Generation of mouse embryos and tissue collection

Generation of *Sox3* transgenic mice by pronuclear injection was described previously [23]. The *Nr* and *Sr* lines were maintained on a C57BL/6×CBA mixed genetic background. *Sr/+;Nr/+* mice/embryos were generated by either of the following crosses: Male *Sr/+;+/+*×Female *+/+;Nr/+* or Male *+/+;Nr/+*×Female *Sr/+;+/+.* For BrdU analysis, pregnant mice were injected 2 h before sacrifice intraperitoneally with 50 mg/kg BrdU (Sigma. cat. No. 59-14-3) dissolved to 10 mg/ml in PBS (pH 7.4). Upon collection, embryos were fixed in 4% paraformaldehyde in PBS, washed 3×10 min in PBS, cryoprotected in 30% sucrose in PBS, embedded in OCT (Tissue Tek) and stored at −80°C. Tissue sections (10–16 µm) were prepared using a Leica CM1900 cryostat.

### *Sr* and *Nr* genotyping

The integration site of *Sox3* transgene has been mapped in *Sr* [23] and *Nr* lines. Primers were designed to amplify across the integration site of *Sr* and *Nr* lines for PCR genotyping. Genomic DNA was extracted from tail/embryo biopsies. The following primers and cycle conditions were used. *Sr* transgenic primers: 5′-ACAGCCTTGTGAGTAGGTATGCTCTTG-3′ and 5′-TGGCTGTGGTAACCATTCATAAGGTAG-3′; *Gapdh* primers: 5′-GGTCGGTGTGAACGGGTGAG-3′ and 5′-CCCTTTTGGCTCCACCCTTC-3′. *Sr* cycle conditions: 95°C 2 min, 95°C 30 s, 60°C 30 s and 72°C 3 min (35 times), 72°C 7 min, 25°C 5 min. *Nr* transgenic/wild type primers: 5′-CTGGGTTAGAGAGCAGCATCC-3′ with either 5′-GAGTGTTGGAGGGGGTTGAG-3′ (transgenic) or 5′-GTCCTACTCCCTCAACACCTGTC-3′ (wild type); and *Sry* primers: 5′-CACTGGCCTTTTCTCCTACC-3′ and 5′-CATGGCATGCTGTATTGACC-3′. *Nr* cycle conditions: 95°C 30 s, 95°C 30 s, 60°C 1 min and 72°C 40 s (35 times), 1 cycle of 72°C 5 min, 1 cycle of 22°C 10 min.

### Quantitative Western blot analysis

10.5 dpc mouse embryos were lysed in 200 µl RIPA buffer (150 mM NaCl, 1.0% Nonident P-40 (NP40), 0.5% deoxycholate, 0.1% SDS, 50 mM Tris-HCl, pH 7.4) supplemented with protease and phosphatase inhibitors (10 mM β glycerol phosphate, 1 mM PMSF, 5 mM NaF, 10 mM sodium vanadate, 1 µg/ml leupeptin and 0.005% v/v aprotinin). 40 µl of 5× SDS loading buffer (312.5 mM Tris, 10% glycerol, 11.5% SDS, pH to 6.8, 0.1% Bromophenol blue, 10% β-mercaptoethanol) was added to the samples. Samples were resolved by 12% SDS-PAGE and subjected to immunoblot analysis using anti-hSox3 (R&D systems, 1∶2500) and anti-β-tubulin (Cell Signalling Technologies, 1∶1000) antibodies. Signals were developed using ECL (West Pico, Pierce) or ECF (Amersham Biosciences) substrates and quantified using the Quantity One software (Bio-Rad). Three embryos for each genotype were analysed.

### qPCR quantitation of *Sox3* transgene copy number

gDNA was isolated from tail tips using a High Pure PCR Template Purification kit (Roche, cat. no.: 11796828001). qPCR was performed on an ABI 7500 StepOne Plus platform using Fast SYBR Green Master Mix (Applied Biosystems, cat. no. 4385610). All qPCR runs consisted of 40 cycles of the following steps: 60°C for 30 s, plate read, 95°C for 3 s. In a single qPCR run, one sample of each genotype was assayed with primers against *Sox1*, *Neurog3* and *Sox3*. Three independent sets of genomic DNA were subjected to qPCR analysis and results are presented as the average of these ± SD. *Neurog3* was used as the reference gene and the relative quantity of *Sox1* for wild type XY was set as 1 and all other genotypes were normalised to this. No genotypes deviated significantly from wild type XY when compared pair-wise using a Student's *t*-test, illustrating that this assay is capable of accurately measuring gene dosage. The relative quantity for *Sox3* versus *Neurog3* was then normalised to a value of one for wild type XY and the relative quantity of *Sox3* for other genotypes was taken as a measure of gene dosage. Estimation of gene dosage did not alter when *Sox1* was used as the reference gene instead of *Neurog3*. Primers used were as follows: *Sox3* (117 bp): 5′- GAACGCATCAGGTGAGAGAAG-3′ and 5′-GTCGGAGTGGTGCTCAGG-3′; *Sox1* (120 bp): 5′- CCCCAGAGACACAACAACCT-3′ and 5′- AGTCACCCACTTCTGCTTCG-3′, *Neurog3* (171 bp): 5′-CCTCTCAGACGGTGGAGTTATATT-3′ and 5′-ATGTTGTACATAGCCTGCAAGTC-3′.

### Histology

For H&E staining, tissue sections were air-dried for 2 h at RT, stained in 0.1% haematoxylin for 20 s, rinsed in water for 5 min, immersed in 0.5% eosin for 15 s, rinsed in water for 5 s, dehydrated in 50% EtOH followed by 70% EtOH for 10 s each, equilibrated in 95% EtOH for 30 s, 100% EtOH for 1 min and histolene for 15 s. Tissue sections were then mounted in xylene based DePex mounting medium Gurr® (Merck, cat. no.: 36125). For Nissl staining, tissue sections were air-dried for 2 h at RT, stained at 0.1% cresyl violet for 5 min and then washed in RO water for 3 min. They were then dehydrated in 95% EtOH for 10 s, 100% for 10 s, equilibrated in histolene for 30 s and mounted in xylene-based DePex mounting medium Gurr® (Merck, cat. no.: 36125).

### Immunofluorescence

Immunofluorescence was performed on cryostat tissue sections as described previously [23]. For BrdU staining, sections were air-dried for 2 h then washed with PBS/0.1% Triton for 10 min, denatured in 2 N HCl at 37°C for 30 min, neutralised in 0.1 M borate buffer (pH 8.5) at RT, washed 3 times in PBS for 10 min and then blocked and immunostained as described previously [23]. Antibodies and dilutions were: goat anti-SOX3 (R&D systems cat. no.: AF2569, 1∶100), rabbit anti-EGFP (Abcam cat. no.: ab290, 1∶1000), rabbit anti-RF (kindly donated by A. Meiniel; 1∶1000; [48]), sheep anti-BrdU (Biodesign International cat. no.: M20107S, 1∶1000), rabbit anti-active Caspase-3 (BD pharmingen cat. no.: 559565, 1∶1000), donkey anti-goat IgG (Jackson ImmunoResearch Laboratories Inc., 1∶400), donkey anti-rabbit IgG Cy3 (Jackson ImmunoResearch Laboratories Inc. cat. no.: 711-165-152, 1∶400), donkey anti-sheep IgG Cy3 (Jackson ImmunoResearch Laboratories Inc. cat. no.: 713-165-147, 1∶400), donkey anti-goat Cy5 (Jackson ImmunoResearch Laboratories Inc. cat. no.: 705-175-147, 1∶400).

### Whole mount and section *in situ* hybridisation

Whole mount and section (10 µm) *in situ* hybridisation was performed as described previously [23]. Probes were transcribed *in vitro* from plasmid templates as follows: *Wnt1* (*Hind*III, T7 polymerase; [49], kindly provided by M. Wassef); *Msx1* (*Bam*HI, SP6 polymerase, a kind gift from Y. Lallemand & C. Ramos); *Fibcd1* (*Spe*I, T3 polymerase, IMAGE clone ID: 6848779); *Bmp6* was amplified from cDNA using the following primers: 5′-GTTCTCCCCACATCAACGAC-3′ and 5′-CCAGCCAACCTTCTTCTGAG-3′ and the product cloned into pGEMT®-T Easy vector system I (Promega, Cat. no.: A1360) (*Spe*I, T7 polymerase).

### Image analysis

Bright field images for whole mount tissue were captured using a Olympus DP70 digital camera mounted on a Nikon SMZ1000 dissecting microscope with AnalySIS software. For tissue sections, a Zeiss Axioplan 2 Imaging upright microscope with Axiovision software was used to obtain immunofluorescence images, while Zeiss Axiophot upright microscope with AnalySIS software was used to obtain bright field images. All captured images were processed by Adobe Photoshop CS. For quantitative BrdU analysis, the following additional procedures were performed. Prior to image capturing, BrdU-positive controls were used to standardise the exposure time and the gain factor of the camera using AxioVision Rel 4.7 software. The medial ganglionic eminence does not have *Sox3* transgene expression and was expected to have similar level of cellular proliferation between wild type and *Sox3* transgenic individuals. Hence, the medial ganglionic eminence served as a reference for BrdU labelling and detection efficiency. For each image of the SCO, an image of the medial ganglionic eminence from the same section was captured using identical exposure time and gain factor. Subsequently, cell counting was performed using ImageJ software [50] without image manipulation and any knowledge of the genotype. For each SCO, three sections separated at an interval of 20 µm were counted. All counts were taken from an average of three repeated counts. For each section, the quantity of BrdU positive SCO cells was scored as the total number of BrdU-positive cells per total number of SCO cells. Similarly, the number of BrdU-positive medial ganglionic eminence cells was scored as the total number of BrdU-positive cells per total number of cells from the selected region of the medial ganglionic eminence. The final proliferative index was obtained by normalising the number of BrdU-positive SCO cells to that of the medial ganglionic eminence from the same section. For data presentation, the proliferative indexes of wild type embryos were set to 1, while those of the *Sox3* transgenic samples were calculated accordingly. Two-tailed unpaired Student's *t*-test was performed for statistical analysis.

### SCO microdissection and RNA extraction

SCO microdissection was performed on stage-matched (53–55 somite) embryos. Embryos were placed on their side and an incision horizontal to the anterior-posterior axis was made above the optic placode. Excised tissue was placed ventral-side down and a V-shaped cut was made to remove the telencephalon followed by the removal of surface ectoderm. A “cup-like” tissue with intact diencephalon and mesencephalon and most dorsal rhombencephalon was left. Small incisions were made at the dorsal midline from both the posterior and anterior end of the cup. This allowed the cup to lay flat on the surface of the microscope stage like a “book”. The PC can be visualised as translucent tracts across the central region of the tissue. The SCO was excised by cutting around the anterior, posterior and lateral limits of the PC. Four SCO from each genotype were pooled into one biological replicate. SCO tissue was stored in RNA*protect* Cell Reagent (Qiagen, no. #: 76526) until used. For RNA extraction, the tissue was homogenised by brief sonication and QIAshredder (Qiagen, cat. no.: 79654) treatment. RNA was extracted using a RNeasy Plus Mini Kit (Qiagen, cat. no.: 74134). RNA quality and concentration was assayed using an Agilent 2100 Bioanalyser.

### qRT-PCR analysis

cDNA was prepared using an Omniscript Reverse Transcription kit (Qiagen, cat. no.: 205110) with 80 ng of RNA, 5.6 µM oligo(dT)_12–18_ (Invitrogen, cat. no.: 18418-012), 1.25 µM dN_6_ (Geneworks, cat. no.: RP-6), 10 U Proctector RNase inhibitor (Roche Applied Science, cat. no.: 03 335 399 001). qRT-PCR was performed with Fast SYBR Green Master Mix (Applied Biosystems, cat. no.: 4385610) on ABI 7500 StepOne Plus platform. All qPCR cycles consisted of 40 cycles of the following steps: 60°C for 30 s, plate read, 95°C for 3 s. Primers used are as follows: *Sox3* (117 bp): 5′- GAACGCATCAGGTGAGAGAAG-3′ and 5′-GTCGGAGTGGTGCTCAGG-3′; *EGFP* (95 bp): 5′-ACGACGGCAACTACAAGACC-3′ and 5′- GTCCTCCTTGAAGTCGATGC-3′, *Msx1* (109 bp): 5′- GCCCCGAGAAACTAGATCG-3′ and 5′- TTGGTCTTGTGCTTGCGTAG-3′, *Wnt1* (103 bp): 5′-TACTGGCACTGACCGCTCT-3′ and 5′-GAATCCGTCAACAGGTTCGT-3′, *Bmp6* (87 bp): 5′-CAAGTCTTGCAGGAGCATCA-3′ and 5′-CCAGCCAACCTTCTTCTGAG3′, *Fibcd1* (101 bp): 5′-CAGCTGGCTTCCAGGTCTAC-3′ and 5′-CCAACCTCGGAAAAAGTTCA-3′, *Gapdh* (143 bp): 5′-ACCCAGAAGACTGTGGATGG-3′ and 5′-ATGCCAGTGAGCTTCCCGTTCAGC-3′; *Sdha* (106 bp): 5′-AACACTGGAGGAAGCACACC-3′ and 5′-GCACAGTCAGCCTCATTCAA-3′. Results were normalised to *Sdha*. Controls for no reverse transcriptase and no template controls were performed for product specificity. Melt curve analysis was undertaken to ensure amplification efficiency.

## Supporting Information

Figure S1 Characterisation of CH phenotype in *Sox3* transgenic mice. A,B: H&E stained sagittal sections of P26 brains showing that the fourth ventricle was not expanded in *Nr/+* CH mice (B) in comparison to wild type (A). C–F: Immunohistochemical analysis of SOX3 (red; C,E) and EGFP (green; D,F) expression in wild type (C,D) and Nr/+ (E,F)18.5 dpc coronally sectioned embryos. Note expression of the endogenous *Sox3* gene and the *Sox3* transgene in the SCO and the slight dysmorphology of the SCO in the *Nr/+* embryo. (D) and (F) are EGFP stains of (C) and (D), respectively. G–I: H&E stained coronal sections of wild type (G), *Nr/+* (H) and *Nr/+* + CH (I) adult brains. SCO hypoplasia was observed in *Nr/+* mice. J,K: Nissl stained sagittal sections of wild type (J) and *Sr/+;Nr/+* (K) 14.5 dpc embryos. SCO dysplasia was evident in *Sr/+;Nr/+* embryos. A small remnant of SCO-like tissue was present in the posterior region (indicated by #). L–Q: Nissl-stained sagittal sections of wild type (L,N,P) and *Sr/+;Nr/+* (M,O,Q) 15.5 dpc (L–O) and 18.5 dpc (P,Q) embryos. (N) and (O) are boxed regions of (L) and (M), respectively. The SCO remnant in *Sr/+;Nr/+* embryos (#) has a similar appearance to wild type (M, O). Both the SCO and pineal gland were missing in *Sr/+;Nr/+* 18.5 dpc embryos. Similar to 14.5 and 15.5 dpc embryos, elongated cells resembling SCO ependymal cells were observed at the very posterior limit of the SCO region of *Sr/+;Nr/+* embryos (#). 4V: fourth ventricle. Cer: cerebellum. EpTh: epithalamus. Asterisk: region of missing pineal gland. Dorsal is towards the top and anterior is towards the left (A,B,G–Q). Scale bars: 500 µm (A,B), 100 µm (C–I, N–O) and 400 µm (J–M, P–Q).
(TIF)

Figure S2 SOX3 expression, apoptosis analysis and ChP morphology in **wild type** and *Sox3* transgenic embryos. A–H: SOX3 immunohistochemical analysis of wild type (A,B,E,F) and *Nr/+* (C,D,G,H) embryos. In wild type embryos, a lower level of SOX3 was evident in the SCO at 15.5 dpc (A,B) and 18.5 dpc (E,F) in comparison to flanking periluminal cells. This difference in SOX3 expression was less obvious in single transgenic (*Nr/+*) embryos (C,D,G,H). I–P: Sagittal sections showing the absence of SCO cell apoptosis in wild type (I,J,M,N) and *Sr/+;Nr/+* (K,L,O,P) embryos at 12.5 dpc (I,K) and 13.5 dpc (M,O). Arrowheads indicate non-specific staining. (J), (L), (N) and (P) are DAPI stains of (I), (K), (M) and (O), respectively. Q–T: Nissl-stained sagittal sections of wild type (Q,S) and *Sr/+;Nr/+* (R,T) 15.5 dpc embryos showing similar histology of ChP from the lateral and third ventricles. U–X: Additional BrdU immunofluorescence (sagittal) sections showing elevated cellular proliferation in the SCO region of 12.5dpc *Sr/+;Nr/+* embryos. 3V: third ventricle. LV: lateral ventricle. Dorsal is towards the top and anterior is towards the left (I–P and U–X). Scale bars: 100 µm (A–P and U–X), 25 µm (O–T).
(TIF)
