# Supplementary figures and images for: Congenital Hydrocephalus and Abnormal Subcommissural Organ Development in Sox3 Transgenic Mice
Source: PLoS One. 2012 Jan 26;7(1):e29041. doi: 10.1371/journal.pone.0029041 (PMC3266892; doi:10.1371/journal.pone.0029041)

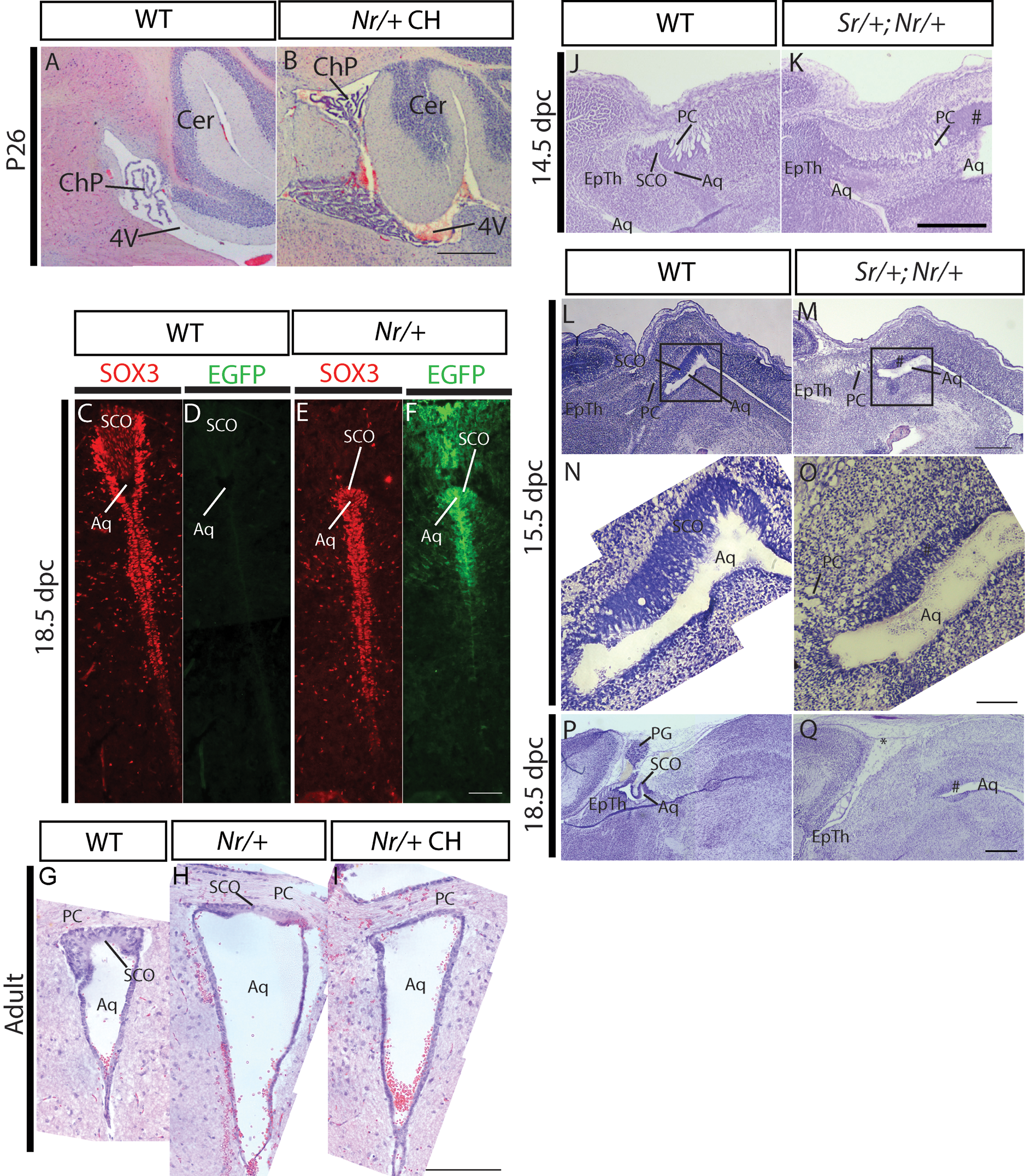

Supplement: Figure S1 — Characterisation of CH phenotype in Sox3 transgenic mice. A,B: H&E stained sagittal sections of P26 brains showing that the fourth ventricle was not expanded in Nr/+ CH mice (B) in comparison to wild type (A). C–F: Immunohistochemical analysis of SOX3 (red; C,E) and EGFP (green; D,F) expression in wild type (C,D) and Nr/+ (E,F)18.5 dpc coronally sectioned embryos. Note expression of the endogenous Sox3 gene and the Sox3 transgene in the SCO and the slight dysmorphology of the SCO in the Nr/+ embryo. (D) and (F) are EGFP stains of (C) and (D), respectively. G–I: H&E stained coronal sections of wild type (G), Nr/+ (H) and Nr/+ + CH (I) adult brains. SCO hypoplasia was observed in Nr/+ mice. J,K: Nissl stained sagittal sections of wild type (J) and Sr/+;Nr/+ (K) 14.5 dpc embryos. SCO dysplasia was evident in Sr/+;Nr/+ embryos. A small remnant of SCO-like tissue was present in the posterior region (indicated by #). L–Q: Nissl-stained sagittal sections of wild type (L,N,P) and Sr/+;Nr/+ (M,O,Q) 15.5 dpc (L–O) and 18.5 dpc (P,Q) embryos. (N) and (O) are boxed regions of (L) and (M), respectively. The SCO remnant in Sr/+;Nr/+ embryos (#) has a similar appearance to wild type (M, O). Both the SCO and pineal gland were missing in Sr/+;Nr/+ 18.5 dpc embryos. Similar to 14.5 and 15.5 dpc embryos, elongated cells resembling SCO ependymal cells were observed at the very posterior limit of the SCO region of Sr/+;Nr/+ embryos (#). 4V: fourth ventricle. Cer: cerebellum. EpTh: epithalamus. Asterisk: region of missing pineal gland. Dorsal is towards the top and anterior is towards the left (A,B,G–Q). Scale bars: 500 µm (A,B), 100 µm (C–I, N–O) and 400 µm (J–M, P–Q). (TIF) [file pone.0029041.s001.tif]

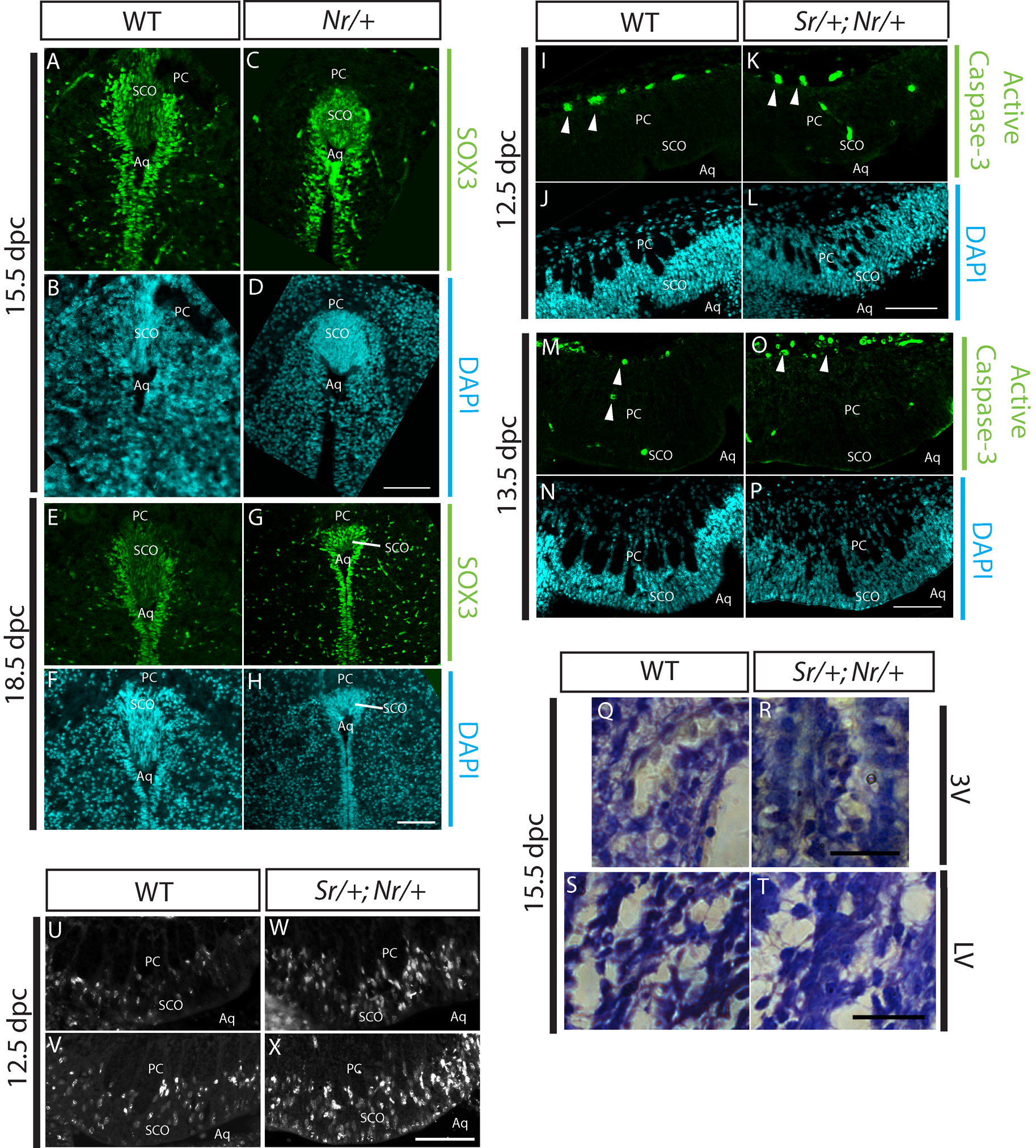

Supplement: Figure S2 — SOX3 expression, apoptosis analysis and ChP morphology in wild type and Sox3 transgenic embryos. A–H: SOX3 immunohistochemical analysis of wild type (A,B,E,F) and Nr/+ (C,D,G,H) embryos. In wild type embryos, a lower level of SOX3 was evident in the SCO at 15.5 dpc (A,B) and 18.5 dpc (E,F) in comparison to flanking periluminal cells. This difference in SOX3 expression was less obvious in single transgenic (Nr/+) embryos (C,D,G,H). I–P: Sagittal sections showing the absence of SCO cell apoptosis in wild type (I,J,M,N) and Sr/+;Nr/+ (K,L,O,P) embryos at 12.5 dpc (I,K) and 13.5 dpc (M,O). Arrowheads indicate non-specific staining. (J), (L), (N) and (P) are DAPI stains of (I), (K), (M) and (O), respectively. Q–T: Nissl-stained sagittal sections of wild type (Q,S) and Sr/+;Nr/+ (R,T) 15.5 dpc embryos showing similar histology of ChP from the lateral and third ventricles. U–X: Additional BrdU immunofluorescence (sagittal) sections showing elevated cellular proliferation in the SCO region of 12.5dpc Sr/+;Nr/+ embryos. 3V: third ventricle. LV: lateral ventricle. Dorsal is towards the top and anterior is towards the left (I–P and U–X). Scale bars: 100 µm (A–P and U–X), 25 µm (O–T). (TIF) [file pone.0029041.s002.tif]
